# Single-cell mtDNA dynamics in tumors is driven by coregulation of nuclear and mitochondrial genomes

**DOI:** 10.1038/s41588-024-01724-8

**Published:** 2024-05-13

**Authors:** Minsoo Kim, Alexander N. Gorelick, Ignacio Vàzquez-García, Marc J. Williams, Sohrab Salehi, Hongyu Shi, Adam C. Weiner, Nick Ceglia, Tyler Funnell, Tricia Park, Sonia Boscenco, Ciara H. O’Flanagan, Hui Jiang, Diljot Grewal, Cerise Tang, Nicole Rusk, Payam A. Gammage, Andrew McPherson, Sam Aparicio, Sohrab P. Shah, Ed Reznik

**Affiliations:** 1 Tri-Institutional PhD Program in Computational Biology & Medicine, Weill Cornell Medicine, New York City, NY USA; 2 Computational Oncology, Department of Epidemiology and Biostatistics, Memorial Sloan Kettering Cancer Center, New York City, NY USA; 3 Human Oncology and Pathogenesis Program, Memorial Sloan Kettering Cancer Center, New York City, NY USA; 4 Department of Molecular Oncology, British Columbia Cancer Research Centre, Vancouver, British Columbia Canada; 5 Institute of Cancer Sciences, University of Glasgow, Glasgow, UK; 6 CRUK Beatson Institute, Glasgow, UK

**Keywords:** Cancer, Genomics

## Abstract

The extent of cell-to-cell variation in tumor mitochondrial DNA (mtDNA) copy number and genotype, and the phenotypic and evolutionary consequences of such variation, are poorly characterized. Here we use amplification-free single-cell whole-genome sequencing (Direct Library Prep (DLP+)) to simultaneously assay mtDNA copy number and nuclear DNA (nuDNA) in 72,275 single cells derived from immortalized cell lines, patient-derived xenografts and primary human tumors. Cells typically contained thousands of mtDNA copies, but variation in mtDNA copy number was extensive and strongly associated with cell size. Pervasive whole-genome doubling events in nuDNA associated with stoichiometrically balanced adaptations in mtDNA copy number, implying that mtDNA-to-nuDNA ratio, rather than mtDNA copy number itself, mediated downstream phenotypes. Finally, multimodal analysis of DLP+ and single-cell RNA sequencing identified both somatic loss-of-function and germline noncoding variants in mtDNA linked to heteroplasmy-dependent changes in mtDNA copy number and mitochondrial transcription, revealing phenotypic adaptations to disrupted nuclear/mitochondrial balance.

## Abstract (web summary)

Amplification-free single-cell whole-genome sequencing shows that genomic, evolutionary and biophysical factors collectively drive cell-to-cell variation in mitochondrial DNA copy number.

## Main

Tumors commonly accumulate mutations and copy number alterations to mitochondrial DNA (mtDNA)^1,2^. The functional effects of these genetic changes on cell metabolism^3,4^, apoptotic potential^5,6^, innate immunity^7^ and other phenotypes depend on at least the following two key factors: the fraction of mutated mitochondrial genomes in the cell (heteroplasmy) and the total number of mtDNAs in the cell (mtDNA copy number)^8,9^. Furthermore, because mtDNA mutations normally arise over the course of human development, somatic cell division, aging and tumorigenesis, mtDNA genotypes are nonrandomly distributed across cells and consequently display potentially large cell-to-cell variation^2,10,11^.

The prevalence of intracellular and intercellular variability in mtDNA genotype represents both a critical confounder to the characterization of phenotypes associated with mtDNA mutations and an effective cell-endogenous mutational barcode for tracing ongoing somatic evolution^12^. To date, several techniques such as single-cell RNA sequencing (scRNA-seq) and single-cell transposase-accessible chromatin sequencing (scATAC-seq) have been applied to measure mtDNA genotypes across tumors, focusing exclusively on the detection of somatic mutations (as opposed to mtDNA copy number) for use as cell-endogenous lineage markers^13–16^. These methods typically require DNA amplification or other approaches to library preparation that inhibit accurate quantification of the absolute mtDNA copy number in a single cell. Yet, the total number of wild-type mtDNA copies, which is determined jointly by heteroplasmy and the total mtDNA copy number, is a key property for understanding the genotype-phenotype map of pathogenic mtDNA mutations^8,17,18^. A comprehensive understanding of mtDNA genotypic variability, evolution and functional consequences therefore requires joint measurement of genotype and absolute copy number.

We previously developed a single-cell whole-genome sequencing (scWGS) platform called Direct Library Prep (DLP+) to study genome plasticity, cell-to-cell variation and clonal evolution driven by copy number alterations of the nuclear genomes of human cancers and model systems^19–21^. Because DLP+ is amplification-free and mtDNAs exist in multiple copies within each cell, it uniquely enables the simultaneous, high-fidelity interrogation of mtDNA genotype, mtDNA copy number and nuclear DNA (nuDNA) genotype across single cells. Here we analyzed DLP+ data of 72,275 single cells from engineered breast epithelial cell lines, patient-derived xenograft models of triple-negative breast cancer (TNBC) and high-grade serous ovarian cancer (HGSC) and primary HGSC tumors. Through the application of computational methods to this unique collection of single-cell genomes, we interrogated the regulatory architecture that quantitatively connects single-cell variation in mitochondrial and nuclear genotypes to downstream phenotypes.

## Results

### Per-cell mtDNA copy number quantification by DLP+

**Fig. 1:**
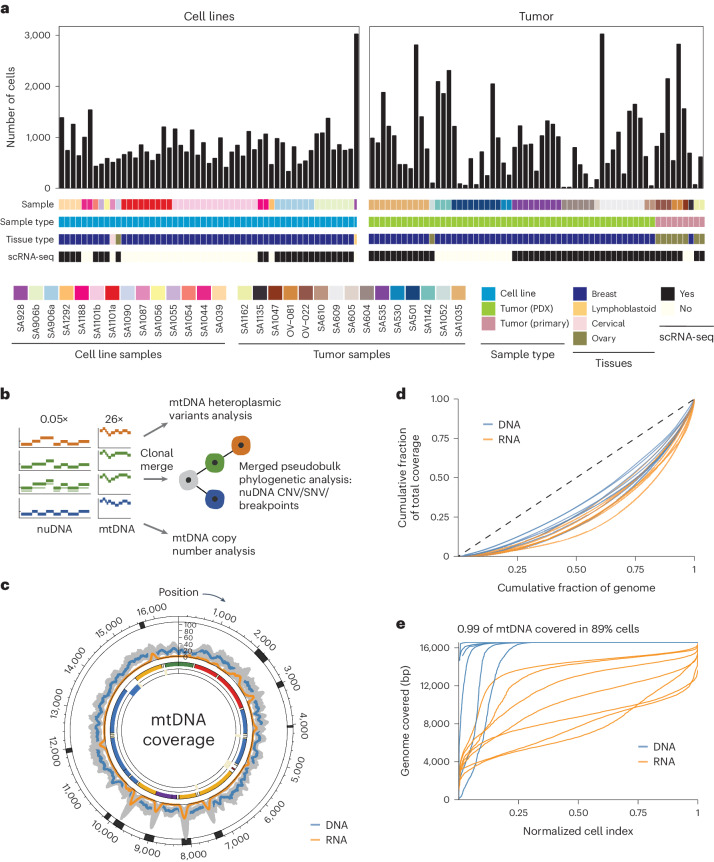
Overview of the data and coverage information. **a**, Summary of the data with histogram indicating the number of cells per DLP+ library corresponding to the samples, annotated by the data type and tissue type. **b**, Schematic representation of DLP+ showing the ability to capture both mtDNA sequence for heteroplasmic variant analysis and absolute mtDNA copy number, along with phylogenetic analysis based on the nuclear genome copy number profile. **c**, Circos plot of the median read coverage across all the samples on a linear scale with the upper and lower bands representing the first and third quartiles. Coverage for DLP+ is in blue, and coverage for scRNA-seq is in orange. The outer track indicates the regions where the coverage breadth is higher in 3′-end scRNA-seq than in DLP+. The mtDNA genes are annotated and colored by mitochondrial complexes. **d**, Lorenz curve with cumulative proportion of total genome coverage on the y-axis and the cumulative proportion of bases on the x-axis. Each curve for DLP+ and 10× scRNA-seq was plotted using the seven PDX genomes. **e**, Depth-of-coverage curves for DLP+ and 10× scRNA-seq for the same seven PDX genomes.

To study mtDNA copy number and heteroplasmy jointly at single-cell resolution, we collected scWGS (DLP+) libraries from a variety of distinct biological settings covering nontransformed cell lines, patient-derived xenografts (PDXs) and primary human tumors (Fig. 1a,b and Supplementary Table 1). These data included previously published^19–21^ sequencing of cell lines from (1) GM18507 diploid lymphoblastoid cell line (*n* = 3,203 cells), (2) nontransformed 184-hTERT mammary epithelial cell line (*n* = 4,011 cells), (3) four *TP53*^−/−^ 184-hTERT cell lines (*n* = 30,012 cells), (4) engineered *TP53*^−/−^;*BRCA2*^+/−^ 184-hTERT cell line (*n* = 2,012 cells), (5) two *TP53*^−/−^;*BRCA2*^−/−^ 184-hTERT cell lines (*n* = 1,056 cells), (6) *TP53*^−/−^;*BRCA1*^+/−^ 184-hTERT cell line (*n* = 463 cells) and (7) *TP53*^−/−^;*BRCA1*^−/−^ 184-hTERT cell line (*n* = 430 cells), as well as the ovarian cancer cell line OV2295 (*n* = 573 cells), cervical cancer cell line HeLa (*n* = 507 cells) and HER2+ breast cancer cell line T-47D (*n* = 2,534 cells). Furthermore, our dataset included 12 different PDX models of TNBC (*n* = 23,466 cells), three of which were cisplatin-treated (*n* = 7,300 cells), one HGSC PDX (*n* = 38 cells) and five primary HGSC tumors (*n* = 4,150 cells) including two newly sequenced surgical resections for a total of 32 distinct samples. For 18 of these samples (eight cell line samples, seven PDX samples and three primary tumor samples), matching scRNA-seq from the same sample was available. Many samples include multiple sequencing libraries performed at different time points as part of a serial passaging experiment, resulting in 127 distinct libraries (median 507 cells per library).

**Extended Data Fig. 1:**
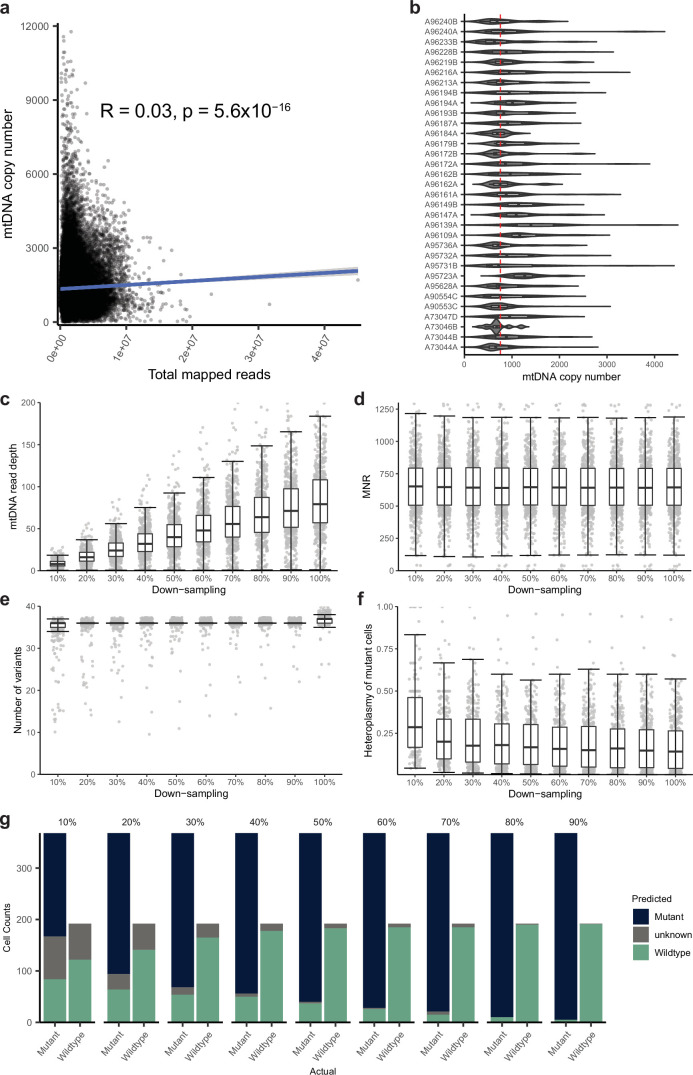
Robust single-cell quantification of mtDNA copy number based on DLP+. **a**, Scatter plot of mtDNA copy number against total mapped read counts for each cell. Two-sided Pearson correlation between mtDNA copy number and total mapped reads results in a correlation coefficient of 0.03, *P* < 5.6 × 10^−16^. Gray-shaded areas represent error bands indicating the 95% confidence interval, and the blue line indicates the regression line. **b**, Per-cell mtDNA copy number estimation of GM18507 lymphoblastoid cells across 33 libraries with at least 15 cells (n = 2,281). All boxplots represent the median, 25th percentile and 75th percentile, and whiskers correspond to 1.5 times the interquartile range. **c**, Downsampling experiment of a SA1090 (OV2295 cell line) library (n = 573 cells) showing a gradual decrease in mtDNA read depth from right to left. All boxplots in the downsampling experiment represent the median, 25th percentile and 75th percentile, and whiskers correspond to 1.5 times the interquartile range. **d**, MNR across all levels of downsampling shows a very consistent MNR around 647 (two-sided, two-sample Wilcoxon test against the original library; all P > 0.78). **e**, Boxplot of the number of variants detected across all levels of downsampling. Total of 37 variants were detected in the original 100% sequencing library. On the other hand, the downsampling resulted in a very stable number of variants at 36 across all levels. The number of variants drastically varied only when the library was down-sampled to 10%. **f**, Distribution of heteroplasmy level of one heteroplasmic variant, m.15500G>A, that was at low heteroplasmy level in the original 100% sequencing depth across all levels of downsampling. The median heteroplasmy was consistent with the original until 30%, below which we saw the median heteroplasmy increase with many cells dropping out. Also, more cells exhibited discrete levels of heteroplasmy due to lower sequencing depth. All boxplots represent the median, 25th percentile and 75th percentile, and whiskers correspond to 1.5 times the interquartile range. **g**, For the same variant, m.15500G>A, the breakdown of the mutant status assignment of the cells with the original 100% sequencing depth is as the ground truth. At 30% of the original sequencing depth, we start to classify true mutant cells as wild-type cells with sensitivity of 0.85 and specificity of 1.

We first compared the coverage of mtDNA in DLP+ and matching 3′-enriched 10× scRNA-seq. Reads aligning to mtDNA were abundant across all cells and, in contrast to the scRNA-seq, covered the entire mitochondrial genome (Fig. 1c). In total, 93.96% of the mitochondrial genome had higher coverage in DLP+ compared to scRNA-seq, enabling comparatively robust mtDNA variant calling and mtDNA copy number estimation directly from primary DLP+ sequencing data. Read depth per cell and the relative capture efficiency of mtDNA/nuDNA had a low correlation, suggesting minimal technical bias derived from sequencing depth in calculating mtDNA copy number (*R* = 0.03, *P* < 10^−15^; Extended Data Fig. 1a). Notably, DLP+ data were both broader and deeper compared to scRNA-seq data from the same sample in seven PDX (Kolmogorov–Smirnov test, all *P* < 2.2 × 10^−16^; Fig. 1d,e).

Unlike most other single-cell DNA sequencing technologies, DLP+ does not use pre-amplification, enabling relatively unbiased quantification of both nuclear and mtDNA copy numbers at single-cell resolution. Following prior work^2,22^, we estimated mtDNA copy number by comparing the read depth of mtDNA- and nuDNA-aligned reads and calibrating mtDNA ploidy to the baseline ploidy of nuDNA. Lymphoblastoid GM18507 cells typically contained 756 copies of mtDNA per cell (25th and 75th percentiles: 575 and 999, respectively) with highly robust and reproducible mtDNA copy number estimates across sequencing libraries (Extended Data Fig. 1b). In silico downsampling of one of the libraries deeply sequenced with a median mtDNA read depth of 79× per cell to a median mtDNA read depth of 8× per cell indicated stable estimation of mtDNA copy number and heteroplasmic mtDNA variant calling down to 30% of the original sequencing depth (Extended Data Fig. 1c–g). The presence of ~1,000 copies of mtDNA per cell is consistent with lower-throughput digital droplet polymerase chain reaction estimates of single-cell mtDNA copy number^23^. Together with the reproducibility of such estimates across sequencing libraries, these analyses establish DLP+ as a robust high-throughput assay for single-cell mtDNA copy number quantification.

### mtDNA copy number correlates with cell size

**Fig. 2:**
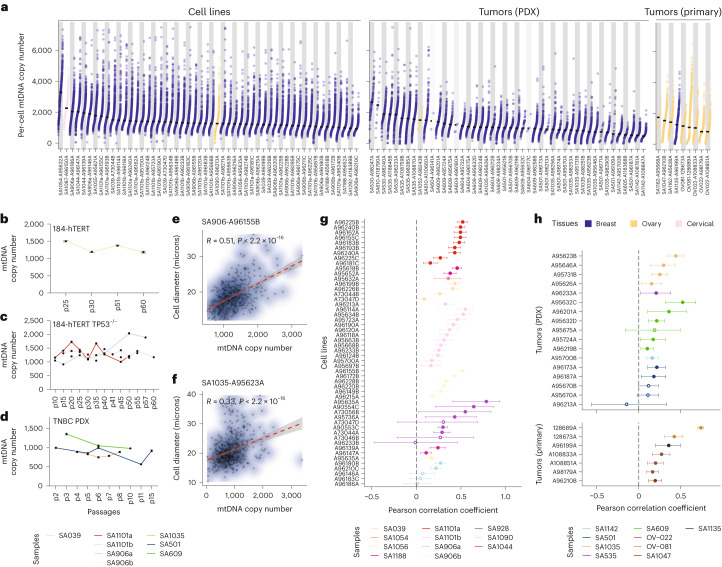
Relative increase in mtDNA copy number with larger cell size. **a**, Distribution of single cell mtDNA copy number across all the cell lines, PDX model and tumor libraries—ordered by decreasing median mtDNA copy number. Within each library, there is a wide variation in the distribution of mtDNA copy numbers. **b**, Median mtDNA copy number of 184-hTERT SA039 cell line across time points p25, p30, p51 and p60 (*n* = 4,011 cells). Error bars indicate the s.e. of the median values. **c**, Same as **b** but for the following four different *TP53*^−/−^ 184-hTERT cell lines—SA906a at time points p10, p15, p25, p30, p40, p50 and p57 (*n* = 4,750 cells), SA906b at time points p20, p30, p35, p40, p45, p50 and p55 (*n* = 6,633 cells), SA1101a at time points p10, p15, p20, p25, p30, p35, p40, p45 and p50 (*n* = 6,720 cells) and SA1101b at time points p15, p20, p25, p30, p35, p40, p41, p45, p50, p55 and p60 (*n* = 11,909 cells). **d**, Same as **c** but for the following three different TNBC PDX samples—SA1035 at time points p4, p5, p6, p7 and p8 (*n* = 3,243 cells), SA609 at time points p3, p6 and p10 (*n* = 2,401 cells) and SA501 at time points p2, p5, p6, p11 and p15 (*n* = 2,153 cells). **e**, Scatter plot showing a positive Pearson correlation (*R* = 0.51, *P* = 1.54 × 10^−43^) between mtDNA copy number and cell diameter for a sequencing library of a *TP53*^−/−^ 184-hTERT SA906b cell line, A96155B (*n* = 627 cells). Contours represent a two-dimensional kernel density estimate. Gray-shaded areas represent error bands indicating the 95% confidence interval, and the red dashed line indicates the regression line. **f**, Same as **e** but for a Pearson correlation (*R* = 0.33, *P* = 1.16 × 10^−19^) on a sequencing library of a TNBC SA1035 PDX, A95623A (*n* = 704 cells). **g**, Dot-and-whisker plot of the Pearson correlation coefficient for every library across all the cell lines (*n* = 52). Consistent, positive coefficient estimation across multiple libraries of the same sample corroborates the relationship between cell size and mtDNA copy number. The dots have their colors filled in only if the library is statistically significant (false discovery rate (FDR) < 0.05). Error bars indicate 95% confidence intervals. **h**, Same as **g** but for tumors (*n* = 17 and 7 libraries for PDXes and tumors, respectively).

**Extended Data Fig. 2:**
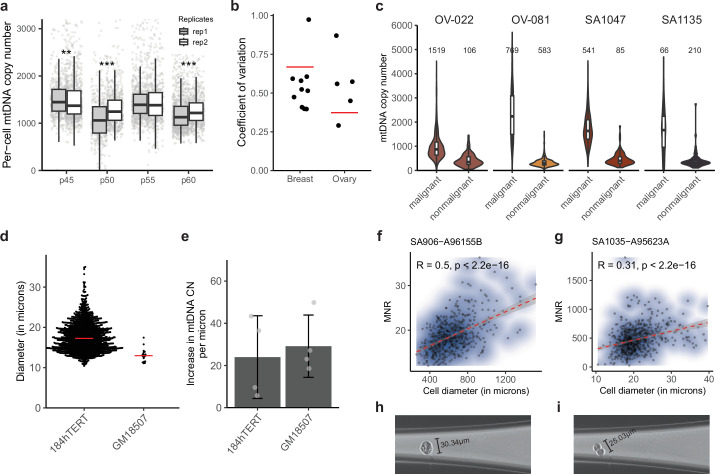
mtDNA copy number represents biophysical and genomic cellular energy demand. **a**, Boxplots showing the distribution of mtDNA copy number across technical replicates over four different time points. *P*-values from the two-sided, two-sample Wilcoxon test are indicated above. The boxplot represents the median, 25th percentile and 75th percentile, and whiskers correspond to 1.5 times the interquartile range. ** denotes *P* < 0.01, *** denotes *P* ≤ 0.001 and no annotation denotes *P* > 0.1. **b**, Coefficient of variation in mtDNA copy number across cells in TNBC (n = 10) and HGSC (n = 5) samples. Red lines indicate a coefficient of variation across breast and ovary bulk tissue tumor samples in PCAWG. **c**, Violin plot of the per-cell mtDNA copy number across malignant and nonmalignant cells across four primary tumors with sufficiently high nonmalignant cells (OV-022: n = 1,625 cells, OV-081: n = 1,352 cells, SA1047: n = 626 cells, SA1135: n = 276 cells, all *P* < 10^−16^). Two-sided, two-sample Wilcoxon test indicates that malignant cells have a significantly higher mtDNA copy number compared to nonmalignant cells across all four tumor samples. The boxplot represents the median, 25th percentile and 75th percentile, and whiskers correspond to 1.5 times the interquartile range. **d**, Distributions of cell diameter in microns for GM18507 (n = 18 cells) and 184-hTERT diploid cells (n = 1,152 cells). **e**, Coefficient for the diameter term in the linear regression model of mtDNA copy number against cell diameter for diploid 184-hTERT and GM18507 cells only (n = 4 libraries each). Error bars represent SD of the coefficient values. **f**, A scatter plot showing a positive two-sided Pearson correlation between MNR and cell diameter for a sequencing library of a TP53^−/−^ 184-hTERT SA906b cell line, A96155B. Gray-shaded areas represent error bands indicating the 95% confidence interval, and the red dotted line indicates the regression line. **g**, Same as **f** but for a sequencing library of a TNBC SA1035 PDX, A95623A. **h**, Microscopic image of a true tetraploid cell from a *TP53*^−/−^ 184-hTERT breast epithelial cell library, SA906-A96228B, taken during cell dispensing as part of the DLP+. The cell size is slightly larger than diploid cells in the same library. **i**, Microscopic image of a doublet from the same library. Although the ploidy is estimated as tetraploid, there are actually two diploid cells sequenced together.

We analyzed DLP+ sequencing data of treatment-naive samples along with 3,203 GM18507 diploid lymphoblastoid cells, included in several DLP+ runs as controls for nuDNA copy number estimation, for a total of 55,930 cells (Methods; Supplementary Table 1). Median copy number across these diverse cells varied from 531 in the TNBC PDX model SA1142 to 3,274 in the *BRCA1*^−/−^*;TP53*^−/−^ 184-hTERT cell line sample SA1054 (Fig. 2a). Per-cell mtDNA copy number estimates were reproducible across technical replicates and exhibited a high degree of temporal stability across multiple 184-hTERT cell lines (Fig. 2b–d and Extended Data Fig. 2a). In contrast to population-level stability in mtDNA copy number, cell-to-cell variation in any single library was substantial (Fig. 2a). Most libraries exhibited a typical coefficient of variation of 0.65, consistent with observations in embryos and parathyroid^24,25^ and the per-sample variation observed in Pan-Cancer Analysis of Whole Genomes (PCAWG) bulk whole genomes of the corresponding cancer type^2^ (Extended Data Fig. 2b).

Next, we used mtDNA copy number quantification from DLP+ to interrogate cell-type-specific mtDNA copy number levels in both malignant and nonmalignant cells from the tumor microenvironment. Prior analysis of mtDNA copy number levels in tumors has focused on comparing estimates of mtDNA copy number from bulk tumor sequencing to matched adjacent-normal tissue^1^, potentially conflating changes in cellular composition with tumor-cell-intrinsic adaptations in mtDNA copy number. In four primary HGSC tumors with DLP+, we were able to identify both malignant and nonmalignant (corresponding to a mixture of stromal and immune) cells on the basis of nuDNA copy number profiles and found that malignant cells displayed a significantly higher mtDNA copy number (log_2_(fold change) = 1.3–3.0; all *P* < 10^−14^; Extended Data Fig. 2c). These data indicate that mtDNA copy number is elevated in tumor cells relative to colocalized nontumor cells in TNBC and HGSC. Further conclusions from this analysis, however, are limited by the inability to definitively distinguish nontransformed cells of a common cell-of-origin to HGSC from other nontransformed cells such as immune cells.

As mitochondria provide anabolic substrates for both cellular maintenance and proliferation^26–29^, we hypothesized that cell-to-cell variation in mtDNA copy number within clonally related cells may reflect bona fide variation in the energetic and anabolic demands of single cells^30,31^. Such changes in anabolic demand might, for example, result from normal variation in cell size, which has been previously posited in the literature and recently quantified in budding yeast^24,25,32^. We analyzed coregistered bright field images from the DLP+ platform (*n* = 4,011 184-hTERT breast epithelial cells, *n* = 26,024 of eight 184-hTERT-derived cell lines and *n* = 1,731 GM18507 diploid lymphoblastoid cells) and correlated estimates of cell size from these images with single-cell mtDNA copy number. The diameter of diploid cells ranged from 10.43 µm to 50.38 µm and varied significantly according to lineage (Extended Data Fig. 2d). This corresponded to an approximate 24 and 29.2 mtDNA copy number increase per micron, respectively (Extended Data Fig. 2e). In total, 46/52 sequencing libraries (covering 11 distinct cell lines) demonstrated a statistically significant positive correlation between cell size and mtDNA copy number (Pearson correlation, *Q* < 0.05; Fig. 2e,g), corroborating previous studies in budding yeast^27,32,33^. We then studied tumor cells, analyzing 5,476 images of cells across 40 libraries of six TNBC PDX models and 4,005 images across nine libraries of five primary HGSC samples. In total, 20/24 sequencing libraries showed a significant positive correlation between cell diameter and mtDNA copy number (Fig. 2f,h). We also correlated the mtDNA-to-nuDNA ratio (MNR), that is, the number of copies of mtDNA per average haploid nuclear genome, against cell diameter, and found statistically significant results across conditions (Extended Data Fig. 2f,g). These findings confirm that, in both cultured cells and human tumors, cell-to-cell variation in mtDNA copy number is associated with a biophysical adaptation in cell size.

### Stoichiometric adaptation of mtDNA copy number to whole-genome doubling (WGD)

**Fig. 3:**
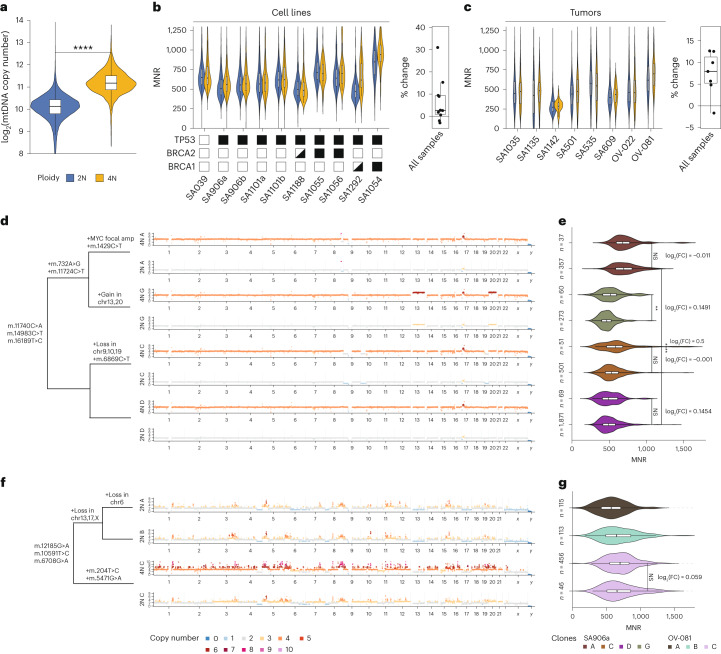
MNR homeostatically balances with WGD but exhibits clone-specific differences. **a**, Distribution of per-cell mtDNA copy number of all the diploid (*n* = 42,351) and tetraploid (*n* = 6,269) cells, colored by nuDNA ploidy. Two-sided, two-sample Wilcoxon test indicates that diploid cells have a significantly different mtDNA copy number compared to tetraploid cells (*P* < 10^−16^). Boxplots represent the median, 25th percentile and 75th percentile, and whiskers correspond to 1.5 times the interquartile range. **** denotes *P* ≤ 0.0001. **b**, Left, violin plots of per-cell MNR between diploid and tetraploid cells of all the cell lines derived from 184-hTERT breast epithelial cells (*n* = 10 samples). Filled and half-filled boxes indicate homozygous and heterozygous loss-of-function genotypes, respectively. Right, boxplot showing the distribution of percent change in MNR from diploid to tetraploid cells. All boxplots represent the median, 25th percentile and 75th percentile, and whiskers correspond to 1.5 times the interquartile range. Bottom, the haplotype-specific states. **c**, Same as **b** but for PDX models and primary tumor samples (*n* = 8 samples). **d**, nuDNA copy number profile of *TP53*^−/−^ 184-hTERT cell line SA906a for diploid and tetraploid cells of clones A, C, D and G (*n* = 3,219 cells). Colors correspond to copy number states. A phylogenetic tree with major mutational events in both genomes labeled is on the left. amp, amplification. **e**, Violin plots of MNR for each of the clones, split into diploid and tetraploid cells. Diploid and tetraploid cells of clones A, C and D indicate NS differences in MNR (two-sided Wilcoxon test). Clone G shows a significant difference (two-sided Wilcoxon test), but the FC is small. All boxplots represent the median, 25th percentile and 75th percentile, and whiskers correspond to 1.5 times the interquartile range. ** denotes *P* < 0.001 and **** denotes *P* ≤ 0.0001. **f**, Same as **d** but for clones A, B and C in a primary HGSC sample, SPECTRUM-OV-081 (*n* = 730 cells). Clone C has both diploid and tetraploid cells. **g**, Same as **e** but for a primary HGSC sample, SPECTRUM-OV-081. There is no significant difference in the MNR of diploid and tetraploid cells of clone C (two-sided Wilcoxon test). NS, not significant; FC, fold change.

**Extended Data Fig. 3:**
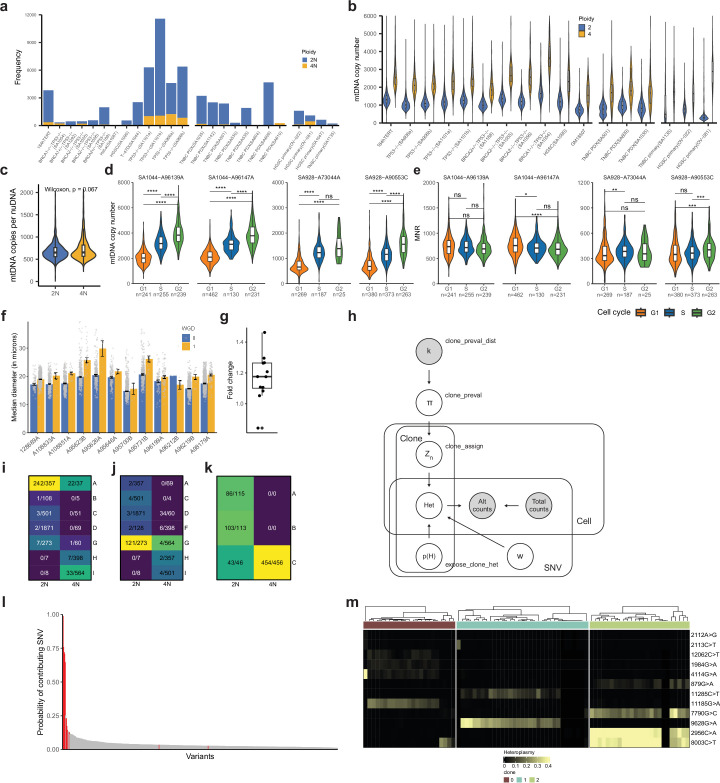
Within-clone analysis of mtDNA-nuDNA ratio in response to whole-genome doubling. **a**, Total number and proportion of diploid and tetraploid cells plotted for each sample. **b**, Comparison of mtDNA copy number between diploid and tetraploid cells across all 9 184h-TERT cell lines and 7 tumor samples as well as GM18507 lymphoblastoid cells (two-sided, two-sample Wilcoxon test, all *P* < 7.7 × 10^−9^). All boxplots represent the median, 25th percentile and 75th percentile, and whiskers correspond to 1.5 times the interquartile range. **c**, Violin plot of MNR in diploid (n = 3,475) and tetraploid (n = 350) cells across all four 184-hTERT breast epithelial cell lines. There is no significant difference between the two groups (two-sided, two-sample Wilcoxon test, *P* = 0.067). All boxplots represent the median, 25th percentile and 75th percentile, and whiskers correspond to 1.5 times the interquartile range. **d**, Boxplot of the mtDNA copy number distribution of cell cycle-sorted cells in different phases, G1, S and G2, across two sequencing libraries of T-47D breast cancer cell line, SA1044-A96139A (n = 735 cells) and SA1044-A96147A (n = 823 cells) and of lymphoblastoid cell line, SA928-73044A (n = 481 cells) and SA928-A90553C (n = 1,016 cells). All boxplots represent the median, 25th percentile and 75th percentile, and whiskers correspond to 1.5 times the interquartile range. Pairwise significance is indicated by two-sided Wilcoxon tests. **e**, Same as **d**, but for MNR. **f**, Bar plot of the median diameter, measured in microns, for both diploid and tetraploid cells for each library across the 12 sequencing libraries of tumor samples. **g**, Boxplot of the median, 25th percentile and 75th percentile of fold change of median diameter for tetraploid cells over diploid cells across the same 12 sequencing libraries in **f**. The whiskers correspond to 1.5 times the interquartile range. **h**, Graphical model of MityBayes. MityBayes takes raw counts of the alternate allele and total depth per cell across mtDNA variants. It infers the clonal assignment of the cells based on clone-specific heteroplasmy level and weighing of informative variants. **i**, Heatmap indicating the presence of mtDNA variant, m.1429C>T. Each cell indicates a fraction of mutant cells out of the total number of cells corresponding to diploid and tetraploid across clones in *TP53*^−/−^ 184-hTERT sample, SA906a. This variant is present in both diploid and tetraploid cells of clone A. **j**, Same as **i** but for m.6869C>T in *TP53*^−/−^ 184-hTERT sample, SA906a. This variant is present in both diploid and tetraploid cells of clone G. **k**, Same as **i** but for m.6708G>A in SPECTRUM-OV-081 sample. This variant is present in both diploid and tetraploid cells of clone C. **l**, Ranking of the weight variable that indicates the probability of the variant contributing to the clonal assignment is plotted in a descending order. The real variants were colored in red. **m**, Heatmap of heteroplasmy across clones determined from mtDNA variants. The mtDNA variants-based clonal labels are on the top.

We hypothesized that somatic alterations in the nuclear genome, and especially large-scale changes to total copy number might contribute to the extensive variation in mtDNA copy number observed in Fig. 2a. In particular, we anticipated that WGD events, which have previously been associated with large metabolic changes and increase in cell sizes and are common in TNBC and HGSC, may be major contributors to mtDNA copy number variation in any given sample^34,35^. WGD was a readily identifiable and frequent event in DLP+ data—we observed WGD in an average of 13% of all sequenced cells from cell lines, 4.7% of all cells from sequenced PDXs and 18% of all cells from sequenced primary tumors (Extended Data Fig. 3a). Interestingly, there was only a small difference in the number of mtDNA variants in diploid and tetraploid cells (Supplementary Table 4). On the other hand, tetraploid cells had significantly higher mtDNA copy numbers than diploid cells across all cell lines, PDX models and primary tumor samples (two-sample Wilcoxon test, all *P* < 10^−15^; Fig. 3a and Extended Data Fig. 3b).

Because coordinated transcription between the nuclear and mitochondrial genomes is necessary for proper stoichiometric assembly of respiratory complexes^36–38^, we tested whether the mtDNA copy number would increase in direct proportion to the ploidy of the nuclear genome^39^ (Methods). To do so, we investigated how MNR varies in tetraploid versus diploid populations of related subclones in a common sequencing library. In parental 184-hTERT cells, the MNR difference between diploid and tetraploid cells was negligible (log_2_(fold change) = 7.5 × 10^−3^, *P* = 0.067; Extended Data Fig. 3c). Similar marginal differences in MNR were observed between tetraploid versus diploid cells in most 184-hTERT-derived lines, with the exception of *BRCA1*-null 184-hTERT cells that exhibited 31% (SA1292) and 15% (SA1054) increases in MNR in tetraploid cells relative to diploid cells (one-sided Wilcoxon test, *P* = 9.3 × 10^−4^ and *P* = 2.4 × 10^−7^, respectively; Fig. 3b). We observed a similar tendency for preservation or small increases in MNR in tetraploid cells relative to diploid cells in the majority of eight PDX and primary tumor samples with sufficient tetraploid/diploid cells for analysis (percent change −6.3% to 12.6%; 2/8 samples statistically significant: one-sided Wilcoxon test, both *P* < 0.025; Fig. 3c). These data establish that for the majority of samples, tetraploid cells and diploid cells derived from a common progenitor contain roughly equal numbers of mtDNA copies per haploid genome. This dosage homeostasis between mtDNA and nuDNA is remarkable especially because mtDNA replication has generally been thought to be uncoupled from the nuDNA replication^40,41^, however, recent studies suggest additional preferential mtDNA replication during S phase^42,43^. Through fluorescence-activated cell sorting (FACS)-based isolation of cells from T-47D breast cancer and GM18507 lymphoblastoid cell lines, we investigated the relationship between absolute mtDNA copy number and nuDNA ploidy across cell cycle phases and found that the mtDNA copy number was preferentially replicated in the S phase. This resulted in a higher mtDNA copy number, but not MNR, in the G2 and S phases compared to the G1 phase (Extended Data Fig. 3d,e). Additionally, while the mtDNA copy number was approximately doubled in the presence of a WGD, the increase in cell diameter was not as pronounced, indicating that MNR homeostasis is not completely explained by adaptations in cell size (Extended Data Fig. 3f,g). Together, these results suggest that a combination of passive and active mechanisms homeostatically coordinate absolute mtDNA copy number and nuDNA ploidy. We subsequently focused on investigating the factors driving exceptions to this phenomenon.

To better understand why some samples exhibited large increases in MNR in tetraploid cells relative to diploid cells, we investigated in detail the *TP53*^−/−^ 184-hTERT sample SA906a and the primary HGSC tumor SPECTRUM-OV-081, both of which demonstrated large, statistically significant differences in tetraploid versus diploid MNR (9.35% and 12.6%, respectively; one-sided Wilcoxon test, both *P* < 1.2 × 10^−4^). We hypothesized that, in these samples, high levels of clonal diversification produced clones with distinct MNR that could indirectly produce an apparent difference between MNR in diploid and tetraploid cells. To test this hypothesis, we ran HDBSCAN^44^ to detect clusters of cells with similar nuDNA copy number profiles and assigned each cell to a specific clone. Somatic mtDNA variants determined to be informative based on a Bayesian clonal assignment model were present in both diploid and tetraploid cells of the same clones, confirming the presence of both diploid and tetraploid cells within a clone (Methods; Extended Data Fig. 3h–k). Consistent with our hypothesis, we observed substantial differences in the MNR of clone A in SA906a, which is primarily distinguished by the presence of a *MYC* focal amplification, compared to ancestral clone D (log_2_(fold change) = 0.5, Wilcoxon test, *P* < 2.2 × 10^−16^). Notably, diploid and tetraploid cells in the same clone demonstrated indistinguishable MNRs, whereas the differences in MNR between ploidy-matched diploid cells across clones A and D were large and statistically significant (log_2_(MNR) of 0.5, *P* < 10^−15^; Fig. 3d,e). A similar effect was observed in the primary tumor sample SPECTRUM-OV-081, where the clonal differences in MNR (for example, in exceptionally high MNR in clone C) dominated intraclone differences in diploid and tetraploid cells (Fig. 3f,g). These data indicate that clonal diversification can drive apparent differences in MNR between diploid and tetraploid cells, and when clonal identity is controlled, diploid and tetraploid cells demonstrate equivalent MNR levels.

### High MNR increases interferon (IFN) response and depletes hypoxic gene expression

**Fig. 4:**
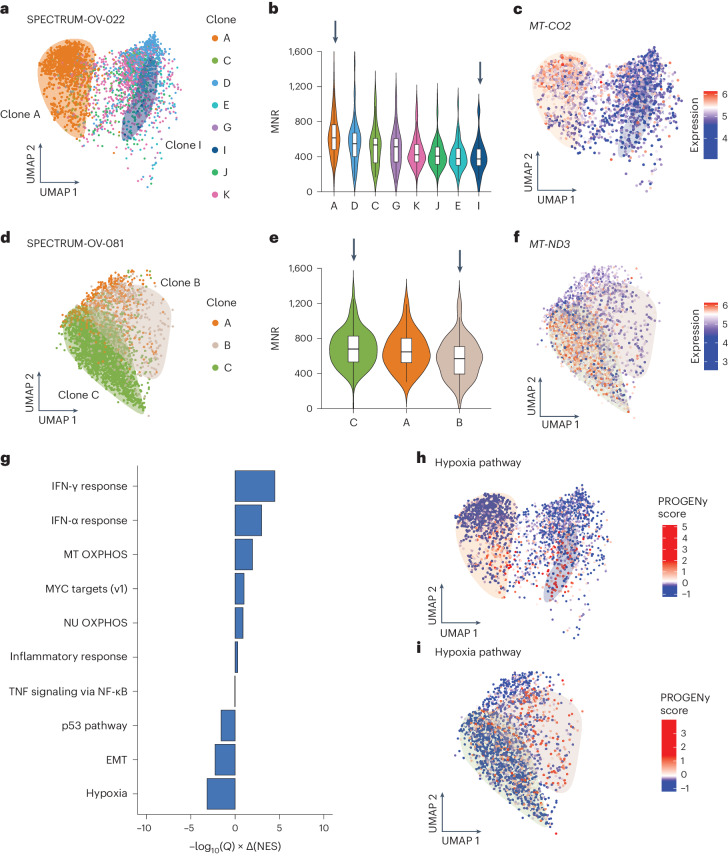
Mapping clones between DLP+ and scRNA-seq reveals that high MNR leads to enrichment in IFN signaling and depletion in the hypoxia pathway. **a**, UMAP of scRNA-seq data from SPECTRUM-OV-022, colored by clones inferred from TreeAlign, *n* = 2,954 cells. Shaded areas demarcate clones A and I, with each encompassing cells of the same clone. **b**, Violin plot of per-cell MNR distribution across eight clones (*n* = 958 cells). Arrows refer to clones A and I having the highest and the lowest median MNR values. All boxplots represent the median, 25th percentile and 75th percentile, and whiskers correspond to 1.5 times the interquartile range. **c**, UMAP plot, colored by expression level of *MT-CO2*, which indicates clone A having the highest expression. Shaded areas demarcate clones A and I, with each encompassing cells from the same clone. **d**, Same as **a** but for SPECTRUM-OV-081, *n* = 2,666 cells. Shaded areas demarcate clones B and C, with each encompassing cells of the same clone. **e**, Violin plot of per-cell MNR distribution across three clones (*n* = 700 cells). Arrows refer to clones C and B having the highest and the lowest median MNR values. All boxplots represent the median, 25th percentile and 75th percentile, and whiskers correspond to 1.5 times the interquartile range. **f**, UMAP plot, colored by expression level of *MT-ND3*, which indicates clone C having the highest expression. Shaded areas demarcate clones B and C, with each encompassing cells from the same clone. **g**, Differential expression of MSigDB hallmark gene sets for tumor samples, between clones with the highest and the lowest MNR. Differential expression is quantified by directional −log_10_(*Q*): here >0 denotes upregulation in clones with high MNR; <0 denotes downregulation. EMT, epithelial-mesenchymal transition. **h**, UMAP plot colored by PROGENy score for hypoxia pathway, which indicates a depletion in clone A. Shaded areas demarcate the two clones—A and I, with each encompassing cells from the same clone. **i**, UMAP plot colored by PROGENy score for hypoxia pathway, which indicates a depletion in clone C. Shaded areas demarcate the two clones—B and C, with each encompassing cells from the same clone. TNF, tumor necrosis factor; NF-κB, nuclear factor kappa B.

**Extended Data Fig. 4:**
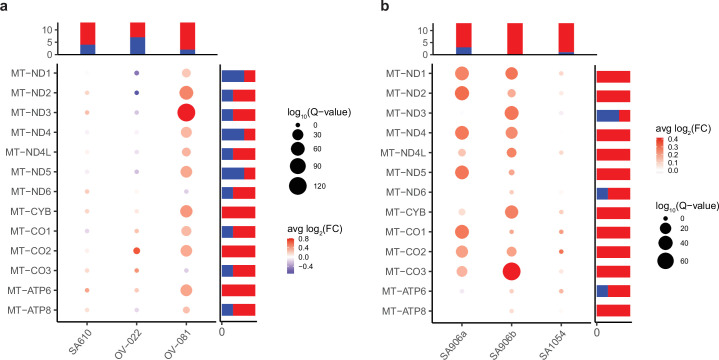
Transcriptional phenotype of high MNR cells compared against low MNR cells in tumors. **a**, Differential expression of mtDNA-encoded genes between clones with the highest and the lowest MNR across the tumor samples based on Wilcoxon rank sum test. Colors indicate the average log_2_ fold change, while the dot size indicates the log_10_ of adjusted p-values. **b**, Same as **a** but for engineered 184-hTERT cell lines.

We next asked if clone-specific differences in MNR elicited phenotypic consequences. We computationally assigned cells in scRNA-seq to clones identified from matched DLP+ using TreeAlign^45^ across samples with both DLP+ and matching scRNA-seq data (Methods). We then compared mtDNA-encoded gene expression patterns of clones with the highest MNR to those with the lowest MNR. For instance, HGSC primary tumor SPECTRUM-OV-022 contained eight clones that closely clustered clones (A, C, D, E, G, I, J and K; Fig. 4a,b). Clone A, which had a high MNR, had higher expression of mtDNA-encoded *MT-CO2* compared to clone I, which had the lowest MNR (Fig. 4c). A similar pattern was observed in SPECTRUM-OV-081, which showed three clones in the UMAP—clones A, B and C (Fig. 4d,e). Clone C (highest MNR) had higher *MT-ND3* expression compared to clone B (lowest MNR; Fig. 4f). We then expanded this analysis across all tumors, comparing tumor subclones for cases with large clonal differences in MNR (log_2_(MNR) > 0.15). For each of the three tumor samples with sufficiently large differences in MNR across clones, we compared the transcriptional profiles of cells in clones with the maximal and minimal MNR (including one PDX and two primary HGSC tumors), observing that transcription of mtDNA-encoded genes was significantly higher in MNR-high clones compared to MNR-low clones (Extended Data Fig. 4a). Similarly, we observed enrichment in mtDNA-encoded gene expression for MNR-high clones for cell lines (Extended Data Fig. 4b). While an association between MNR and mtDNA expression has been suggested in earlier work^30,46^, these data directly connects subclonal variation in MNR to mtDNA-encoded gene expression.

To more granularly understand the association between MNR and non-mtDNA-encoded gene expression, we undertook a pathway enrichment analysis. Pathway analysis on the three tumor samples with matched DLP+ and scRNA-seq using differential expression between high and low MNR clones identified 8/51 Molecular Signatures Database (MSigDB) hallmark gene sets with recurrent enrichment/depletion, including elevated expression of mtDNA oxidative phosphorylation (OXPHOS) pathway and innate immune-related pathways (Fig. 4g). Interestingly, only SPECTRUM-OV-022 exhibited statistically significant enrichment in nuDNA-encoded OXPHOS in the same direction as mtDNA-encoded OXPHOS, ruling out MNR as a dominant regulator of nuDNA-encoded OXPHOS transcription. Instead, high MNR clones exhibited a recurrent depletion in hypoxic gene expression—in both SPECTRUM-OV-022 and SPECTRUM-OV-081, high MNR clones (OV-022 clone A; OV-081 clone C) had significantly lower PROGENy hypoxia enrichment score than low MNR clones (SPECTRUM-OV-022 clone I; SPECTRUM-OV-081 clone B; Wilcoxon test; OV-022, *P* = 0.0064, OV-081, *P* = 7.9 × 10^−6^; Fig. 4h,i). Variation in MNR in vivo is thus primarily associated with changes to mtDNA-encoded, but not nuclear-DNA-encoded, OXPHOS expression, as well as transcriptional adaptations to nuDNA-encoded metabolic pathways.

### Dosage-dependent mtDNA variant effects on mtDNA copy number

**Fig. 5:**
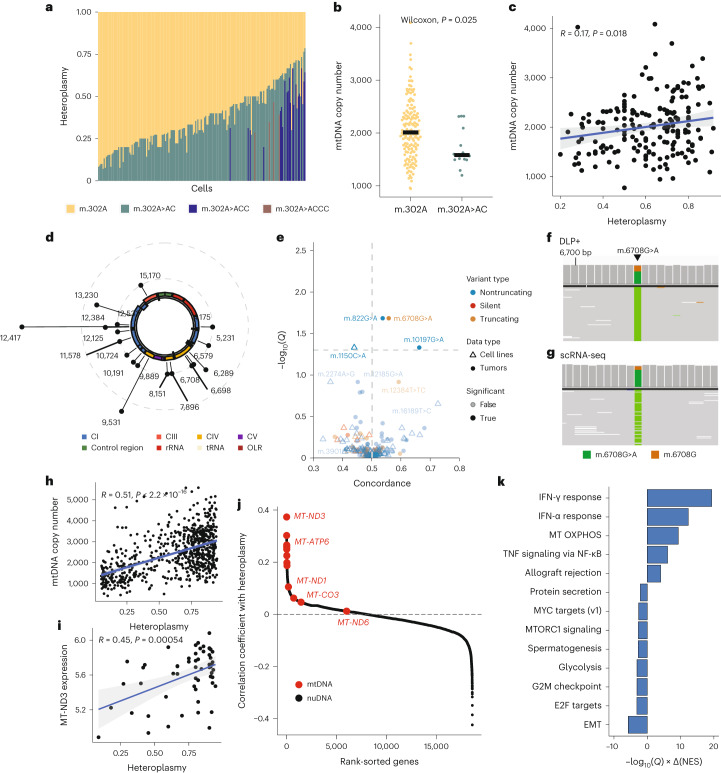
Genetic perturbation in mtDNA can elicit a dosage-dependent decrease in mtDNA copy number. **a**, Length heteroplasmy composition of m.302 across 126 single cells in HGSC primary tumor sample, SA1047. Each bar represents a cell. **b**, Effect of length heteroplasmy composition, the reference (*n* = 148 cells) and the major allele (*n* = 14 cells), at m.302 on mtDNA copy number. Reported *P* value from two-sided, two-sample Wilcoxon signed rank test. **c**, Scatter plot showing heteroplasmy of the reference allele at m.302 and mtDNA copy number (two-sided Pearson correlation, *R* = 0.17, *P* < 0.02). Gray-shaded areas represent error bands indicating the 95% confidence interval, and the blue line indicates the regression line. **d**, Circos plot with lollipop indicating the genomic position of truncating variants identified in DLP+ across both cell lines and PDX models (CI, CIII, CIV, CV, control region, rRNA, tRNA, OLR). **e**, Concordance between the heteroplasmy level of the truncating, silent and nontruncating variants and the mtDNA copy number across all cell lines and tumors. **f**, Integrative Genomics Viewer^59^ of m.6708G>A substitution in DLP+ sequencing data of SPECTRUM-OV-081 Infracolic Omentum sample. The stacked histogram on top reflects the distribution of the reads supporting the reference and the alternate alleles. **g**, Same as **f** but for scRNA-seq of the same sample at the matched site. **h**, Two-sided Pearson correlation between mtDNA copy number and heteroplasmy level of the m.6708 truncating variant in SPECTRUM-OV-081 in DLP+ (*R* = 0.51, *P* = 2.9 × 10^−48^). Gray-shaded areas represent error bands indicating the 95% confidence interval, and the blue line indicates the regression line. **i**, Two-sided Pearson correlation between *MT-ND3* gene expression and heteroplasmy level of the same m.6708G>A truncating variant in SPECTRUM-OV-081 in matched scRNA-seq (*R* = 0.45, *P* = 0.00054). Gray-shaded areas represent error bands indicating the 95% confidence interval, and the blue line indicates the regression line. **j**, Pearson correlation coefficient between the heteroplasmy level of the truncating variant m.6708 and expression of all the genes in the SPECTRUM-OV-081 sample. The mtDNA-encoded genes are colored in red. **k**, Enrichment of MSigDB hallmarks gene sets for tumors using the rank-sorted gene list based on correlation coefficient, quantified by directional −log_10_(*Q*): here >0 denotes upregulation in cells with high heteroplasmy truncating variants and <0 denotes downregulation. CI, mitochondrial complex I; CIII, mitochondrial complex III; CIV, mitochondrial complex IV; CV, mitochondrial complex V; rRNA: ribosomal RNA; tRNA, transfer RNA; OLR, light strand origin of replication.

Recently, a genome-wide association study (GWAS) of variation in mtDNA copy number in whole blood reported that certain germline mtDNA insertions, including those affecting the length of a homopolymeric block at m.302 associated with the balance of mtDNA replication and transcription, could potentially regulate mtDNA copy number^47–49^. Interestingly, this study revealed (using scATAC-seq) that individual cells from the same patient often exhibited different heteroplasmic levels of such insertions, suggesting that cell-to-cell variation in mtDNA genotype could, in *cis*, drive variation in mtDNA copy number levels. Because of the unique ability of DLP+ to simultaneously track mtDNA copy number and genotype in phenotypically distinct tumor cells, we evaluated if DLP+ could identify the length heteroplasmy at m.302 and, if so, test the hypothesis that the length heteroplasmy is associated with changes in single-cell mtDNA copy number. For each of the 32 samples in our dataset, we genotyped the mtDNA of individual cells (Methods) and identified cells with homopolymeric insertions at m.302. We identified a single sample (SA1047) with a sufficient number of cells for subsequent analysis (at least 20 diploid cells with minimum coverage of 10 reads at position 302; Fig. 5a). Considering diploid cells only to avoid any confounding effects associated with nuDNA, we quantified both mtDNA copy number and the heteroplasmy of the reference allele (m.302A) and evaluated the association between the two. This analysis revealed that, consistent with the bulk GWAS data, cells with the reference allele (m.302A) demonstrated elevated mtDNA copy number (Wilcoxon test, *P* = 0.025; Fig. 5b), and m.302A heteroplasmy was associated with higher mtDNA copy number (Pearson correlation, *R* = 0.17 and *P* = 0.018; Fig. 5c), indicating that mtDNA genotype itself may modulate mtDNA copy number levels.

We next analyzed truncating mutations in mtDNA, which arise in approximately 20% of all cancers and are thought to impair mitochondrial respiration^2,7,50^. Prior studies have suggested that somatic truncating mtDNA mutations can also elicit increases in mtDNA copy number^51–53^. However, because prior analyses were undertaken from bulk sequencing data, there remains little understanding of the adaptive response of mtDNA copy number to single-cell variation in heteroplasmy. Analysis of mtDNA copy number and truncating mtDNA mutations in 11,691 total cells across seven distinct cell lines, five PDX samples and five primary tumor samples identified 23 truncating mutation events spanning across 19 distinct genomic positions and 20 silent mutations spanning across 20 distinct genomic positions (truncating variants shown in Fig. 5d). Consistent with prior reports^7,54^, these mutations predominantly affected complex I subunits at homopolymeric hotspots (for example, m.12417).

Among the 23 truncating variants across both cell lines and tumors, we identified a statistically significant association between the heteroplasmy of m.6708G>A (encoding a complex IV truncating mutation) and mtDNA copy number (*Q* = 2.1 × 10^−2^; Fig. 5e). The pathogenicity and clinical significance of this variant have been reported previously in mitochondrial myopathy and rhabdomyolysis^55^, confirming our prediction that somatic truncating mutations in mtDNA can have deleterious effects on the cellular fitness in the form of increased mtDNA copy number. We corroborated the presence of m.6708G>A in matched scRNA-seq data (Fig. 5f,g) and, consistent with the positive correlation between heteroplasmy and mtDNA copy number observed in DLP+ (Fig. 5h), the heteroplasmy of m.6708G>A in scRNA-seq data was positively associated with the expression of mtDNA-encoded genes (Pearson correlation against mtDNA copy number, *P* < 2.2 × 10^−16^; against *MT-ND3* expression, *P* < 0.001; Fig. 5i–k). In contrast, we found no statistically significant association between heteroplasmy and mtDNA copy number levels among 20 silent mutations. Finally, we also evaluated the association between the heteroplasmy of 130 nontruncating mitochondrial variants, the vast majority of which were variants of unknown significance, and mtDNA copy number. This identified two variants (m.822G>A, affecting a nearly universally conserved locus of *MT-RNR1*, and m.10197G>A, a confirmed-pathogenic allele causing Leigh disease^56,57^) whose heteroplasmy significantly associated with elevated mtDNA copy number, implicating these mutations as putative modifiers of resting mtDNA copy number. A fourth variant of unknown significance (m.1150G>A, also affecting *MT-RNR1* and universally conserved in the human germline) was observed in two *TP53*^−/−^ 184-hTERT cell lines and associated with decreased mtDNA copy number. These data establish that single cells adapt to some pathogenic mtDNA mutations, but not silent mutations, by increasing mtDNA copy number in a heteroplasmy/dosage-dependent manner.

## Discussion

Although the few proteins encoded by mtDNA are essential to normal cellular metabolism and physiology, both mtDNA copy number and genotype can vary dramatically across otherwise isogenic populations of cells. Neither the regulatory principles controlling this cell-to-cell variation nor the phenotypes arising from variation to mtDNA copy number in individual cells are well-understood. By applying DLP+ to simultaneously characterize mtDNA copy number, mtDNA genotype and nuDNA genotype in >72,000 cells, we were able to carry out scaled analyses of the biophysical, evolutionary and phenotypic consequences of cell-to-cell variation in mtDNA copy number.

We observed extensive variation in per-cell mtDNA copy number, which is consistent with previous observations^24,25^. By characterizing the quantitative variation in per-cell mtDNA copy number in human cancer in relation to cell size, nuclear ploidy, clonal composition and expression of mtDNA-encoded OXPHOS genes, we have shown that mtDNA copy number variation reflects, at least in part, both anabolic cellular demands for increased levels of cellular building blocks to produce larger cells^58^ and stoichiometric equipoise to ensure appropriate relative levels of mtDNA and nuDNA^36^ (that is, the MNR). Remarkably, the emergence of genetically distinct subclones can perturb MNR levels, and such variation in MNR appears to have specific transcriptional consequences on mtDNA-derived, but not nuDNA-derived, OXPHOS transcription. This represents a previously poorly considered class of phenotypic variation that arises from clonal evolution in cancer with potential implications for improved understanding of cellular fitness.

We also find, in agreement with the population-scale analysis of healthy individuals and patients with cancer, that certain mtDNA genotypes were themselves associated with changes to copy number^47^. Unlike bulk sequencing studies, we harnessed DLP+ to quantitatively interrogate how mutant dosage, or mtDNA heteroplasmy, in individual cells affected mtDNA copy number. We observed that both relatively common germline polymorphisms (at m.302) and highly pathogenic somatic mutations elicited adaptive increases in mtDNA copy number in a heteroplasmy-dependent manner. Given that disruption of different functional components of mtDNA (such as complex I versus complex IV subunits or tRNA genes versus protein-coding genes) is known to produce vastly different phenotypes and sensitively depend on cell-of-origin, investigation of the adaptive mtDNA copy number response to functionally distinct mtDNA mutations in diverse cellular backgrounds may prove insightful. In summary, our work here implicates the coevolution of the mitochondrial and nuclear genomes in individual cells as a regulator of cellular fitness and phenotypic states in cancer.

## Methods

### Experimental model and participant details

#### Cell culture and PDXs

Cell lines were generated as previously described^19,21^. In brief, the samples included (1) an immortalized normal human female breast epithelial cell line 184-hTERT L9, (2) four sets of 184-hTERT cell lines with perturbations in *TP53*^−/−^ passaged over multiple time points, (3) five 184-hTERT cell lines with a variety of genetic perturbations in the repair pathway, including *TP53*^−/−^, *BRCA1*^−/−^, *BRCA2*^+/−^ and *BRCA2*^−/−^ and (4) a GM18507 lymphoblastoid cell line. The samples also included three sets of TNBC PDX models. The University of British Columbia’s Ethics Committees granted approval for all experiments involving human resources. Donors from Vancouver, British Columbia, provided their consent for the Tumor Tissue Repository protocols (TTR-H06-00289, H16-01625). These samples were then transplanted into mice following the Animal Resource Center bioethics protocol (A19-0298-A001), which received approval from both the University of British Columbia’s Animal Care Committee and the BC Cancer Research Ethics Board under protocols H20-00170 and H18-01113. The serial passaging was done by seeding approximately 1 million cells each time and profiled with DLP+ at 4–11 different passage points with a mean of 6,070 cells at each time point.

#### SPECTRUM

All patients from the MSK SPECTRUM cohort^60–62^ provided their consent to the institutional biospecimen banking protocol. The Memorial Sloan Kettering Cancer Center’s Institutional Review Board (IRB) approved all related protocols (15-200 and 06-107). The consent process adhered to the IRB’s standard operating procedures for obtaining informed consent, ensuring that all participants were fully informed and agreed in writing before any study-specific activities commenced. This study was carried out in accordance with the principles of the Declaration of Helsinki and adhered to the Good Clinical Practice guidelines. Matched 10x Genomics 3′-end scRNA-seq and DLP+ were obtained from two patients with HGSC (OV-022 and OV-081). Single-cell suspensions were flow-sorted on CD45 to separate the immune component, and the CD45-negative fractions were then profiled with DLP+.

### Quantification and statistical analysis

#### Mitochondrial variant calling and genotyping

Quality score is assigned to each cell as part of the DLP+ pipeline based on 18 features related to read depth and nuDNA CNV information, as described in ref. ^21^. Only live cells with a quality score of at least 0.75 were kept for further analysis. We developed a single-cell variant calling workflow to identify mtDNA variants in single cells based on our previously described variant calling pipeline^7^. Variants are called by two independent variant-calling pipelines, and only the variants identified by both pipelines were retained for further analysis. The first pipeline is Mutect2 (GATK v4.1.2.0) using the mitochondrial option, which was run on every cell and then merged into a single VCF file. The second pipeline is samtools mpileup (v1.9) to generate a pileup file using variant-supporting reads with a minimum mapping quality (>20) and base quality (>20). This was run on the merged pseudo-bulk of all the single cells for the variant calling step. Variants were required to contain at least two variant-supporting reads in both the forward and reverse directions. PCR duplicates and reads that failed any of the quality checks were removed. As described in ref. ^14^, capturing the agreement of heteroplasmy between the strands is important in eliminating false positive calls. Thus, variants were further filtered based on a high Pearson correlation (*R* ≥ 0.2). Next, the black-listed, homopolymer repeat regions (513–525 and 3105–3109) in the mtDNA genome were filtered out as well^22^. The filtered variants were genotyped by running the second pipeline on individual cells for a per-cell heteroplasmy calculation. Mutational signature and strand bias were assessed as described in ref. ^22,63^. The trinucleotide sequence context (immediate 5′ and 3′) was extracted, and the substitution rate for each context was calculated with the number of substitutions normalized by the frequency of all the observed contexts, in the L and H strand, respectively. We defined the germline variants as variants that enable us to infer the ancestral haplogroup for each cell line. Homoplasmic variants then refer to variants that are not found in the haplogroup of the sample (local private mutations) or in any of the defined haplogroups (global private mutations).

#### Estimation of average nuclear ploidy and baseline ploidy

Both the average ploidy and the baseline ploidy level of each cell were estimated with HMMcopy^64^, as previously described in ref. ^21^. Briefly, for each cell, we calculate the average ploidy as the mean copy number across the 500 kilobase-wide bins in the entire nuclear genome, which is a nonnegative real number. On the other hand, the baseline ploidy of cells is categorized as either diploid, triploid, tetraploid or some other integer value based on the most commonly occurring copy number state across the 500 kilobase-wide bins of the entire nuclear genome.

#### Estimation of mtDNA gross copy number

The mtDNA copy number was calculated for each cell as follows:

$${\rm{mtDNA}}\; {\rm{copy}}\; {\rm{number}}=\frac{{\mathrm{mtDNA}}\,{\mathrm{read}}\,{\mathrm{depth}}}{{\mathrm{nuDNA}}\,{\mathrm{read}}\,{\mathrm{depth}}}\times {\mathrm{average}}\,{\mathrm{ploidy}}$$

The MNR refers to the ratio of mtDNA read depth to nuDNA read depth. Average ploidy was calculated using the mean copy number of all bins across the nuDNA genome from the HMMcopy^64^ result.

#### Determining the cell diameter from microscopic images

DLP+ platform has microscopic image data at the nozzle before the cells are isolated into wells^21^. Microscopic images taken during the dispensing of the cells are used to automatically filter for doublets, and additional manual inspection of tetraploid cell images found that the median number of doublets across 25 sequencing libraries was 3.76%, suggesting that WGD predictions are unlikely to be confounded by doublets (Extended Data Fig. 2h,i and Supplementary Table 3). The diameter was calculated as Waddel disk diameter^21^.

#### A linear regression model for inference of cell size

First, a linear regression model was built to predict mtDNA copy number from the average nuDNA ploidy. Then the model was expanded to a linear multiple regression model to predict mtDNA copy number from cell diameter and the average ploidy. The average ploidy level could deviate from the integer baseline ploidy level in the presence of large chromosomal arm level copy number changes. Benjamini–Hochberg correction was applied for each sequencing library to account for the multiple testing of cells. For plotting, the scale was standardized and normalized to the mean.

#### Comparison of mtDNA copy number across cell cycle phases

Cell cycle analysis was performed on T-47D and GM18507 cell lines generated through the combination of experimental FACS^21^ and PERT^62^ output. FACS cell cycle phase labels were derived by staining cells for their total DNA content using DAPI and then isolating cells into G1-, S- and G2-phase populations before sequencing. PERT was then run on this scWGS data at 500 kb resolution using default model parameters and the FACS labels as initializations for the G1/2- and S-phase populations. PERT calls cells with 5–95% replicated loci as S phase and all others as G1/2 phase. The fraction of replicated loci per cell is also used to scale the total copy number of these cells. Only cells with matching FACS and PERT phase labels were included in the downstream analysis.

#### Relative change in MNR between diploid and tetraploid cells

The change in the MNR was calculated for each group as follows:

$${\mathrm{Difference}}=\frac{{\mathrm{Median}}({\mathrm{MN}}{\mathrm{R}}_{\mathrm{t}})-{\mathrm{Median}}({\mathrm{MN}}{\mathrm{R}}_{\mathrm{d}})}{{\mathrm{Median}}({\mathrm{MN}}{\mathrm{R}}_{\mathrm{d}})}\times 100$$

#### Inference of clones based on nuDNA read counts

Clonal assignment of the cells was done by running HDBSCAN on the two-dimensional embedding from UMAP of the per-cell GC-corrected read count profiles^20^. Parameters used in UMAP and HDBSCAN were the same as previously described—UMAP was run with min_dist = 0.0 and metric = ‘correlation’, whereas HDBSCAN was run with approx_min_span_tree = False, cluster_selection_epsilon = 0.2 and gen_min_span_tree = True.

#### Model description and clonal inference using mtDNA variants

MityBayes is a Bayesian statistical model that systematically assigns cells into clones based on both the presence of mtDNA mutations and their heteroplasmy levels. The inputs to MityBayes are a prior on the number of clones, alternate read counts and the total read counts for each mtDNA variant across the cells. The alternate read counts of a variant in a cell follow a binomial distribution. The total read count at a specific genomic position where a variant is present is equivalent to the number of trials (n) and the clone-specific heteroplasmy level serves as the probability of success (p). Inference is performed using stochastic variational inference in the Pyro package. We generate the variational distributions using the AutoDelta function that uses Delta distributions to construct a MAP guide over the latent space. Optimization is performed using the Adam optimizer. By default, we set a learning rate of 0.1, and the convergence is determined when the relative change in evidence lower bound (ELBO) is lower than 10^−5^. We benchmarked MityBayes against the most similar method available in the literature, MQuad^65^, which does not assign cells to clones based on mtDNA as MityBayes does but rather prioritizes mtDNA mutations that discriminate among different clones. MityBayes weighed the true variants with a higher probability of contribution in the clone assignment and was able to detect the clones when the input variants list was filtered (Extended Data Fig. 3l,m).

#### Integration of scDNA and scRNA data with TreeAlign

TreeAlign was used to computationally integrate scDNA and scRNA data by assigning transcriptional profiles to scDNA-based subclones. Briefly, TreeAlign explicitly models clone-specific copy number dosage effects and defines subclones informed by transcriptional changes from scDNA-based single-cell phylogenies. Here we ran TreeAlign with the following parameters: infer_b_allele = False, repeat = 8, min_clone_assign_prob = 0.9, min_clone_assign_freq = 0.75, min_consensus_gene_freq = 0.55, max_iter = 900, rel_tol = 1e-5, initialize_seed = True, min_cell_count_expr = 40, min_cell_count_cnv = 30, min_gene_diff = 150, min_snp_diff = 60, level_cutoff = 50, min_proceed_freq = 0.80, min_record_freq = 0.75.

#### Pathway enrichment analysis in matched scRNA-seq

CellRanger software (version 4.0.0) was used to perform read alignment, barcode filtering and UMI quantification using the 10× GRCh38 transcriptome (version 3.0.0) for gene expression. Filtered matrices were processed using the Seurat R package (version 3.0.1)^66,67^. The resulting gene-by-cell matrix was log normalized and merged by the patient. Cell-type assignments were computed on each patient with cellassign (version 0.99.2)^68^ using a set of curated marker genes, and cancer cells with a high probability (>0.99) were retained. Clone labels were assigned from using CNV data obtained from DLP+ using CloneAlign (version 0.99.0)^45^. Cell-type annotated matrices for individual patients across time points were integrated with Harmony (version 0.1)^69^ into a single batch-corrected matrix. Dimensionality reduction and visualization as a UMAP embedding were performed with the Seurat R package. Differentially expressed genes (*P* < 0.001, log(fold change) > 0.25) were computed using the Wilcoxon test using clone labels.

#### Concordance between mtDNA copy number and heteroplasmy

Because there are multiple sequencing libraries per sample with cells of different average ploidy, we used a stratified and weighted concordance model to identify pairs of heteroplasmy and mtDNA copy numbers that were consistently associated. Similar to Kendall’s Tau, concordance is a nonparametric measure of correlation that relies on the concept of concordant pairs^70^. The concordance analysis was adapted from ref. ^71^. Briefly, the calculation was done using the concordance function from the survival R package^72^. As with Somers’ D and Kendall’s tau, the magnitude of *c*_scaled_ captures the strength of the effect, with values near −1 or 1 corresponding to strong discordance and concordance, respectively. We weighed each observation by the number of cells in the corresponding library. A *z* score was computed as unscaled concordance minus 0.5 and divided by the square root of the variance, and the resulting value was used to derive a two-tailed *P* value. *P* values were then corrected for multiple testing using the Benjamini–Hochberg method to control the false discovery rate. We filtered for highly covered mtDNA variants with at least ten reads supporting the alternate allele. For each variant, we filtered cells with heteroplasmy less than 0.05 or greater than 0.95 to prevent clusters of cells near 0 or 1 heteroplasmy from erroneously skewing the correlation estimation. Only the variants that had a range of 0.15 were kept for downstream analysis.

### Reporting summary

Further information on research design is available in the Nature Portfolio Reporting Summary linked to this article.

## Online content

Any methods, additional references, Nature Portfolio reporting summaries, source data, extended data, supplementary information, acknowledgements, peer review information; details of author contributions and competing interests; and statements of data and code availability are available at 10.1038/s41588-024-01724-8.

### Supplementary information

Reporting Summary

Supplementary Tables Supplementary Table 1: An overview of the mtDNA DLP+ data. Supplementary Table 2: Per-cell level DLP+ sequencing statistics for 184-hTERT cell lines, HGSC and TNBC tumors. Supplementary Table 3: Summary of doublets and tetraploid cell images across sequencing libraries. Supplementary Table 4: Median number of variants per diploid and tetraploid cells across samples. Supplementary Table 5: Descriptions and prior distributions of random variables and data in the MityBayes model. Supplementary Table 6: Cell cycle dataset with ploidy estimates from the PERT model.

## Data Availability

The sequencing data associated with the study spans already publicly available datasets^19–21^ and are available at the European Genome-Phenome Archive with the accessions EGAS00001006343, EGAS00001004448 and EGAS00001003190. The DLP+ and matching scRNA-seq data for the two patients with HGSC (patient 022 and patient 081) from the MSK SPECTRUM cohort are available via dbGaP (accession phs002857.v2.p1). The processed data are available on Zenodo (10.5281/zenodo.10498240)^73^.

## Code availability

Mutect2 (GATK v4.1.2.0), Samtools (v1.9), CellRanger software (v4.0.0), cellassign (v0.99.2) and CloneAlign (v0.99.0) R packages: R (v4.2.3), Seurat R package (v3.0.1) and Harmony (v0.1) were used in this study. Custom R code to regenerate all figures is available on GitHub (https://github.com/reznik-lab/mtdna-dlp)^74^ with the relevant data and instructions to execute the code.
