# Supplementary material for: Single-cell mtDNA dynamics in tumors is driven by coregulation of nuclear and mitochondrial genomes
Source: Nat Genet. 2024 May 13;56(5):889–99. doi: 10.1038/s41588-024-01724-8 (PMC11096122; doi:10.1038/s41588-024-01724-8)
Supplement: Supplementary file 1 — Reporting Summary [file 41588_2024_1724_MOESM1_ESM.pdf]

nature portfolio

Corresponding author(s): Sohrab P Shah and Ed Reznik

Last updated by author(s): Apr 17, 2024

Reporting Summary

Nature Portfolio wishes to improve the reproducibility of the work that we publish. This form provides structure for consistency and transparency in reporting. For further information on Nature Portfolio policies, see our Editorial Policies and the Editorial Policy Checklist.

Statistics

For all statistical analyses, confirm that the following items are present in the figure legend, table legend, main text, or Methods section.

n/a	Confirmed	
☐	☒	The exact sample size (*n*) for each experimental group/condition, given as a discrete number and unit of measurement
☐	☒	A statement on whether measurements were taken from distinct samples or whether the same sample was measured repeatedly
☐	☒	The statistical test(s) used AND whether they are one- or two-sided<br>*Only common tests should be described solely by name; describe more complex techniques in the Methods section.*
☐	☒	A description of all covariates tested
☐	☒	A description of any assumptions or corrections, such as tests of normality and adjustment for multiple comparisons
☐	☒	A full description of the statistical parameters including central tendency (e.g. means) or other basic estimates (e.g. regression coefficient) AND variation (e.g. standard deviation) or associated estimates of uncertainty (e.g. confidence intervals)
☐	☒	For null hypothesis testing, the test statistic (e.g. *F*, *t*, *r*) with confidence intervals, effect sizes, degrees of freedom and *P* value noted<br>*Give P values as exact values whenever suitable.*
☐	☒	For Bayesian analysis, information on the choice of priors and Markov chain Monte Carlo settings
☒	☐	For hierarchical and complex designs, identification of the appropriate level for tests and full reporting of outcomes
☐	☒	Estimates of effect sizes (e.g. Cohen's *d*, Pearson's *r*), indicating how they were calculated

Our web collection on statistics for biologists contains articles on many of the points above.

Software and code

Policy information about availability of computer code

Data collection	All commercial, open source and custom code software is detailed in the Methods section.
Data analysis	Mutect2 (GATK v4.1.2.0), Samtools (v1.9), CellRanger software (v4.0.0), cellassign (v0.99.2), and CloneAlign (v0.99.0)<br>R packages: R (v4.2.3), Seurat R package (v3.0.1), Harmony (v0.1).<br><br>Custom software that is part of this study is available as specified in the Methods section: R code to regenerate all figures is available on Github (https://github.com/reznik-lab/mtdna-dlp) with the relevant data and instructions to execute the code.

For manuscripts utilizing custom algorithms or software that are central to the research but not yet described in published literature, software must be made available to editors and reviewers. We strongly encourage code deposition in a community repository (e.g. GitHub). See the Nature Portfolio guidelines for submitting code & software for further information.

Data

Policy information about availability of data

All manuscripts must include a data availability statement. This statement should provide the following information, where applicable:

- Accession codes, unique identifiers, or web links for publicly available datasets
- A description of any restrictions on data availability
- For clinical datasets or third party data, please ensure that the statement adheres to our policy

The DLP+ and scRNA-seq data associated with the study spans already publicly available datasets from European Genome-Phenome archive and dbGaP:
- DLP+ data from Laks et al. 2019 is available from EGAS00001006343
- DLP+ data from Salehi et al. 2021 is available from EGAS00001004448
- DLP+ and scRNA-seq data from Funnell et al. 2022 is available from EGAS00001003190
- The MSK SPECTRUM dataset is available via dbGaP (accession number phs002857.v2.p1: http://www.ncbi.nlm.nih.gov/projects/gap/cgi-bin/study.cgi?study_id=phs002857.v2.p1)

The reference file:
- Pre-built reference GRCh38 transcriptome (version 3.0.0) is availabe from 10x Genomics

The processed data are available on Zenodo (https://zenodo.org/records/10498240)

Research involving human participants, their data, or biological material

Policy information about studies with human participants or human data. See also policy information about sex, gender (identity/presentation), and sexual orientation and race, ethnicity and racism.

Reporting on sex and gender	N/A
Reporting on race, ethnicity, or other socially relevant groupings	N/A
Population characteristics	N/A
Recruitment	N/A
Ethics oversight	N/A

Note that full information on the approval of the study protocol must also be provided in the manuscript.

Field-specific reporting

Please select the one below that is the best fit for your research. If you are not sure, read the appropriate sections before making your selection.

☒ Life sciences ☐ Behavioural & social sciences ☐ Ecological, evolutionary & environmental sciences

For a reference copy of the document with all sections, see nature.com/documents/nr-reporting-summary-flat.pdf

Life sciences study design

All studies must disclose on these points even when the disclosure is negative.

Sample size	Cell lines and PDX cohort:<br>For each time series sample, we estimated power to detect clones to 2% for choosing the number of cells to sequence. For number of samples per time series, this was determined due to practical constraints, sampling tumours over a 2.5yr interval. No calculations were used to determine the number of samples/time series.<br><br>Quality-filtered study datasets (MSK SPECTRUM cohort):<br>• scRNA-seq: 2 patients, 5 samples, 2,977 cells
Data exclusions	For all samples, low quality sequenced cells and cells with very few reads mapping to mtDNA were excluded.
Replication	Replicates were performed in the cell lines and the TNBC-SA609 Line 1 Untreated PDX, via one and two 'mixing' experiment respectively to reproduce the observation of fitness clones.<br>Patients with multi-site data had 2-3 samples profiled by DLP+.
Randomization	Randomization was not applicable in this study because the cell line experiments were controlled perturbation experiments, generating time series with or without isogenic TP53 mutation.

Blinding | Blinding was not relevant because the controlled perturbation nature of the experimental design.

Reporting for specific materials, systems and methods

We require information from authors about some types of materials, experimental systems and methods used in many studies. Here, indicate whether each material, system or method listed is relevant to your study. If you are not sure if a list item applies to your research, read the appropriate section before selecting a response.

Materials & experimental systems

n/a	Involved in the study
☒	☐ Antibodies
☐	☒ Eukaryotic cell lines
☒	☐ Palaeontology and archaeology
☐	☒ Animals and other organisms
☒	☐ Clinical data
☒	☐ Dual use research of concern
☒	☐ Plants

Methods

n/a	Involved in the study
☒	☐ ChIP-seq
☒	☐ Flow cytometry
☒	☐ MRI-based neuroimaging

Eukaryotic cell lines

Policy information about cell lines and Sex and Gender in Research

Cell line source(s): Cell lines were generated from the commercially available 184-hTERT cell line as previously described (references [1-4]) and as indicated in the Methods section: "the samples include (1) an immortalized normal human female breast epithelial cell line 184-hTERT L9, (2) four sets of 184-hTERT cell lines with perturbation in TP53-/- passaged over multiple timepoints, (3) five 184-hTERT cell lines with a variety of genetic perturbations in the repair pathway, including TP53-/-, BRCA1-/-, BRCA2+/- and BRCA2-/-,..., and (5) GM18507 lymphoblastoid cell line."

Authentication: These cell lines were used previously and reported in previous papers from our laboratory:
1. Laks, E. et al. Clonal Decomposition and DNA Replication States Defined by Scaled Single-Cell Genome Sequencing. Cell 179, 1207–1221 (2019).
2. Salehi, S. et al. Clonal fitness inferred from time-series modelling of single-cell cancer genomes. Nature 595, 585–590 (2021).
3. Funnell, T. et al. Single-cell genomic variation induced by mutational processes in cancer. Nature (2022) doi:10.1038/s41586-022-05249-0.

Mycoplasma contamination: Mycoplasma testing was negative.

Commonly misidentified lines (See ICLAC register): No commonly misidentified cell lines were used in the study.

Animals and other research organisms

Policy information about studies involving animals; ARRIVE guidelines recommended for reporting animal research, and Sex and Gender in Research

Laboratory animals: The details on mouse strains for xenografting tumours can be found in Section 2 of the Supplementary Information of a previous paper from our laboratory
Reference [1]

Wild animals: These PDX models were reported in previous papers from our laboratory:
1. Salehi, S. et al. Clonal fitness inferred from time-series modelling of single-cell cancer genomes. Nature 595, 585–590 (2021).

Reporting on sex: *Indicate if findings apply to only one sex; describe whether sex was considered in study design, methods used for assigning sex. Provide data disaggregated for sex where this information has been collected in the source data as appropriate; provide overall numbers in this Reporting Summary. Please state if this information has not been collected. Report sex-based analyses where performed, justify reasons for lack of sex-based analysis.*

Field-collected samples: *For laboratory work with field-collected samples, describe all relevant parameters such as housing, maintenance, temperature, photoperiod and end-of-experiment protocol OR state that the study did not involve samples collected from the field.*

Ethics oversight: The Ethics Committees at the University of British Columbia approved all the experiments using human resources. Patients in Vancouver, British Columbia were recruited, and samples were collected under the tumour tissue repository (TTRH06-00289) protocol and transplanted in mice under the Neoadjuvant PDX (University of British Columbia BC Cancer Research Ethics Board H20-00170) protocols.
After informed consent, tumour fragments from patients undergoing excision or diagnostic core biopsy were collected. Tumour

materials were processed and transplanted in mice under the animal resource centre (ARC) bioethics protocol (A19-0298-A001) approved by the animal care committee.

Note that full information on the approval of the study protocol must also be provided in the manuscript.
